# MODERATION OF CUSTODIAL GRANDPARENT’S WELLNESS AND GRANDCHILD'S BEHAVIOR BY RELATIONSHIP QUALITY

**DOI:** 10.1093/geroni/igac059.2398

**Published:** 2022-12-20

**Authors:** Marin Olson, Karly Pyles, Acacia Lopez, Rachel Scott, Danielle Nadorff

**Affiliations:** Mississippi State University, Mississippi State, Mississippi, United States; Mississippi State University, Mississippi State, Mississippi, United States; Mississippi State University, Starkville, Mississippi, United States; Mississippi State University, Starkville, Mississippi, United States; Mississippi State University, Mississippi State, Mississippi, United States

## Abstract

A growing number of grandparents are assuming care of their grandchildren, and both grandparents and children report higher levels of stress than their peers, although the source of this stress remains largely unexplored. In Conger & Donnellan’s Family Stress Model, a parent’s distress can impact the levels of distress experienced by their child, and previous research indicates that parent/child relationship quality impacts children’s mental health. This study extended this to custodial grandfamilies, by investigating whether the relationship quality of custodial grandparents (GP) and grandchildren (GC) would moderate the relation between the health of GC and the wellness of their GP. Participants include 322 custodial grandparents within the United States recruited via Qualtrics Research Panel Survey (m age = 55.66 yr., 86.1% female). Measures included the Achenbach Child Behavior Checklist, Kivnick’s Grandparent Meaning Scale, and grandparents’ wellness. Moderation of relationship quality on the relation between the GP’s wellness and internalizing behaviors of the GC was tested using SPSS’ Process macro. A significant interaction was found (t = 2.31, p = .02) wherein GP’s wellness was not predictive of changes in GC’s behavior for the group with high relationship quality, but greater GP wellness was associated with less GC internalizing behaviors when relationship quality was low (p < .001). These findings indicate that when a GP feels that they have a more positive relationship with their GC, there is a greater likelihood that they will report fewer health concerns and fewer GC’s internalizing behavior symptoms.

